# The Use of Essential Oils from Thyme, Sage and Peppermint against *Colletotrichum acutatum*

**DOI:** 10.3390/plants10010114

**Published:** 2021-01-08

**Authors:** Armina Morkeliūnė, Neringa Rasiukevičiūtė, Lina Šernaitė, Alma Valiuškaitė

**Affiliations:** Laboratory of Plant Protection, Lithuanian Research Centre for Agriculture and Forestry, Institute of Horticulture, LT-54333 Babtai, Lithuania; neringa.rasiukeviciute@lammc.lt (N.R.); lina.sernaite@lammc.lt (L.Š.); alma.valiuskaite@lammc.lt (A.V.)

**Keywords:** biocontrol, chemical composition, inhibition, *Mentha piperita*, *Salvia officinalis*, *Thymus vulgaris*

## Abstract

The *Colletotrichum* spp. is a significant strawberry pathogen causing yield losses of up to 50%. The most common method to control plant diseases is through the use of chemical fungicides. The findings of plants antimicrobial activities, low toxicity, and biodegradability of essential oils (EO), make them suitable for biological protection against fungal pathogens. The aim is to evaluate the inhibition of *Colletotrichum acutatum* by thyme, sage, and peppermint EO in vitro on detached strawberry leaves and determine EO chemical composition. Our results revealed that the dominant compound of thyme was thymol 41.35%, peppermint: menthone 44.56%, sage: α,β-thujone 34.45%, and camphor: 20.46%. Thyme EO inhibited *C. acutatum* completely above 200 μL L^−1^ concentration in vitro. Peppermint and sage EO reduced mycelial growth of *C. acutatum*. In addition, in vitro, results are promising for biological control. The detached strawberry leaves experiments showed that disease reduction 4 days after inoculation was 15.8% at 1000 μL L^−1^ of peppermint EO and 5.3% at 800 μL L^−1^ of thyme compared with control. Our findings could potentially help to manage *C. acutatum*; however, the detached strawberry leaves assay showed that EO efficacy was relatively low on tested concentrations and should be increased.

## 1. Introduction

The strawberry anthracnose can be considered as one of the most important diseases, caused by several species complex of *Colletotrichum* spp.: *Colletotrichum acutatum* J. H. Simmonds, brooks and *C. gloeosporioides* (Penz.) Penz. and Sacc. [1,2]. It also infects and causes diseases in many economically important crops. Strawberry anthracnose causes yield losses of up to 50% and plant death up to 80% and was considered to be a warmer climate zone pathogen, where the optimal temperature is from 15 to 30 °C, with optimal 25 °C temperature [3,4,5]. Strawberry diseases are controlled by several fungicide applications [6,7]. The growing resistance to pesticides and their adverse environmental effects leads to a new environmentally-safe disease control strategy [8,9,10].

Essential oils (EO) demonstrate a distinct level of antimicrobial activity to various ranges of strawberry pathogens [6,9,11,12]. The EO includes terpenes, terpenoids, aromatic, and aliphatic constituents, and most importantly, they contain antioxidants and biologically active compounds.

The EO is commonly described as secondary metabolites with high defence plant effect as they have antimicrobial properties and are non-toxic and biodegradable [13,14,15,16,17]. Harvest dates, storage period, plant extraction method, and climate may affect plant essential oils’ chemical compositions. Several factors that could influence the composition of the EO: climate, geographical location, harvest dates, storage period, and extraction method [18,19].

Plant essential oils are developed commercially on a large scale, most of which are the Lamiaceae family members, including *Thymus. vulgaris*, *Salvia officinalis*, *Mentha piperita* [18,20]. Thyme (*T. vulgaris* L.), sage (*S. officinalis* L.), and peppermint (*M. piperita* L.) EO, as products from plants, have a wide application in pharmacy, fragrance, food industries, however recent studies of essential oils revealed their potential antimicrobial activity [21,22,23]. *S. officinalis* EO affects *Fusarium* spp. growth [15]. *M. piperita* EO inhibits the spread of *Alternaria* spp. and *Fusarium* spp. pathogens [24]. *T. vulgaris* EO has antifungal activity against plant pathogens such as *Monilinia fructicola*, *Botrytis cinerea, Aspergillus flavus* [16,25,26]. *T. vulgaris*, EO can be used as a natural food preservative against casual agents of food-borne diseases like *E. coli*, *Pseudomonas* spp. and others [27].

Plant protection products against plant diseases are necessary to avoid yield and crop losses. However, pesticides have an adverse effect on plants and humans, as they leave residues. The European Green Deal provides a plan to increase environmentally friendly technologies by supporting strategies to reduce pesticides and make agriculture more sustainable. Growing pathogens resistance occurs because of the extensive use of chemical pesticides for plant protection. The new sources of natural active ingredients for plant protection may solve pesticide resistance problems and reduce environmental and food contamination [7,11,12,28]. This study aims to evaluate the inhibition of *C. acutatum* by thyme, sage, and peppermint EO in vitro also on the detached strawberry leaves, and determine EO chemical composition.

## 2. Results

### 2.1. Essential Oils Chemical Composition

The chemical composition of EO is presented in Table 1. In total, 99.33% of thyme and 99.94% of peppermint EO components were identified. Three dominant compounds were determined of thyme: thymol 41.35%, *p*-cymene 16.95%, *γ*-terpinene 10.81%. Peppermint: menthone 44.56%, isomenthone 12.81%, pulegone 10.74%. 99.94% of the total identified compounds in sage EO the highest quantities were set of *α, β*-thujone 34.45%, camphor 20.46%, and eucalyptol 10.33%. 

### 2.2. Essential Oils Antifungal Activity In Vitro

**Figure 1:**
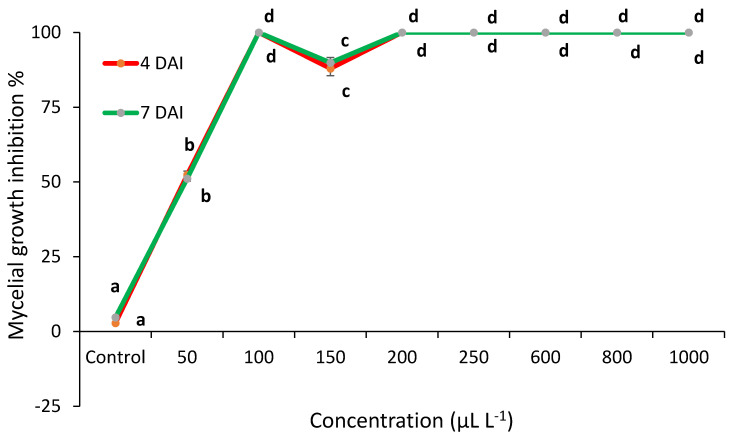
*C. acutatum* mycelial growth inhibition (%) by thyme (*T. vulgaris*) EO at 4 and 7 days after inoculation (4 DAI and 7 DAI). The results presented as means (*n* = 4). The same letter indicates no significant differences between treatments (*p* < 0.05).

EO antifungal activity was assayed at different concentrations on potato dextrose agar (PDA). The inhibition of *C. acutatum* by thyme EO is shown in Figure 1. Thyme EO showed 100% mycelial growth inhibition at 4 and 7 days after inoculation (4 and 7 days after inoculation (DAI)) at 100 μL L^−1^ but did not demonstrate the high antifungal effect at 150 μL L^−1^ at 4 (87.96%) and 7 DAI (89.9%). However, mycelial pathogen growth was inhibited above 200 μL L^−1^.

**Figure 2:**
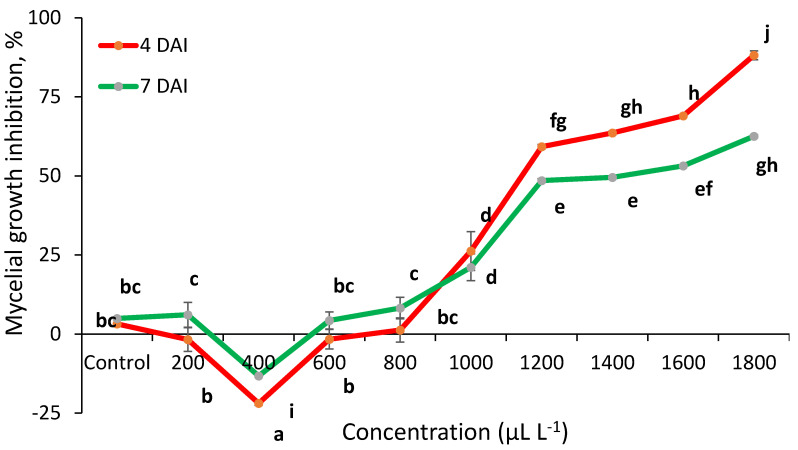
*C. acutatum* mycelial growth inhibition (%) by sage (*S. officinalis*) EO at 4 and 7 days after inoculation (4 DAI and 7 DAI). The results are presented as means (*n* = 4). The same letter indicates no significant differences between treatments (*p* < 0.05).

The mycelial growth inhibition of *C. acutatum* by sage EO is presented in Figure 2. Data indicate that this EO was less effective than thyme. Sage EO showed antifungal activity up to 1000 μL L^−1^ at 4 DAI and achieved the highest effect of 88.14% at 1800 μL L^−1^. However, this EO’s effectiveness at 7 DAI was lower in 1800 μL L^−1^ and reached 62.54%.

**Figure 3:**
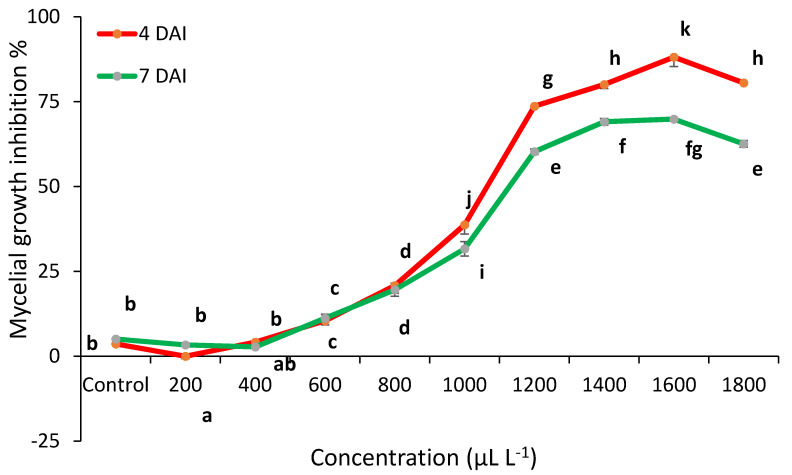
*C. acutatum* mycelial growth inhibition (%) by peppermint (*M. piperita*) EO at 4 and 7 days after inoculation (4 DAI and 7 DAI). The results are presented as means (*n* = 4). The same letter indicates no significant differences between treatments (*p* < 0.05).

The fungicidal activity of peppermint EO against *C. acutatum* is shown in Figure 3. This EO had a similar effect on *C. acutatum* comparing with sage. Meanwhile, peppermint EO reduced the mycelial growth at 600–1800 μL L^−1^ from 20% to 88%. However, the highest antifungal activity was reached at 1600 μL L^−1^ at 4 DAI. 1800 μL L^−1^ EO efficiency decreased to 62.54%.

**Figure 4:**
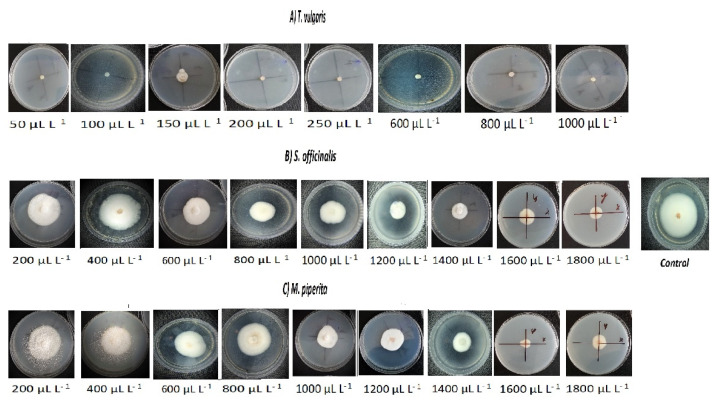
*C. acutatum* mycelial growth inhibition by EO at various concentrations. (**A**) thyme EO; (**B**) sage EO; (**C**) peppermint EO.

*T. vulgaris* EO totally inhibited the mycelial growth at 200–1000 μL L^−1^. The MIC was determined (minimal inhibitory concentration) an equal to 200 μL L^−1^. *S. officinalis* and *M. piperita* EO reduced mycelial growth compared to control. However, this EO was insufficient to inhibit the spread of anthracnose infection (Figure 4) (Detailed information can be found in supplementary materials Tables S1 and S2, Figures S1 and S2). 

### 2.3. Antifungal Activity on Detached Strawberry Leaves

**Figure 5:**
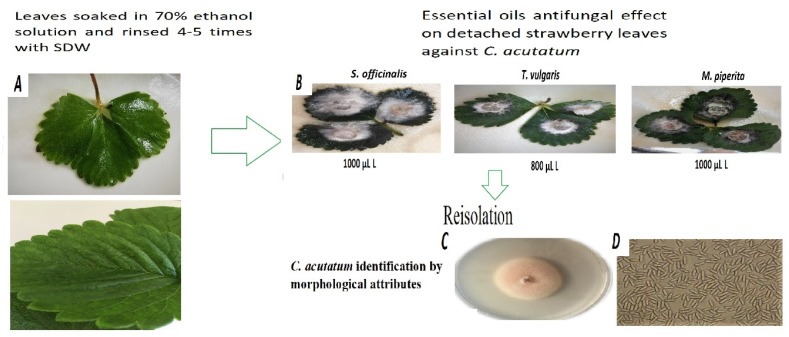
Infection of *C. acutatum* on detached strawberry leaves after EO application. (**A**) Control—not inoculated leaves; (**B**) Leaves treated with EO thyme 800 μL/L, sage and peppermint 1000 μL/L concentrations; (**C**) after reisolation, morphologically confirmed *C. acutatum*; (**D**) after reisolation confirmed *C. acutatum* spores.

The detached strawberry leaf assay was developed to determine the efficiency of essential oils against *C. acutatum* (Figure 5). The results revealed that among all the investigation treatments, only 1000 μL L^−1^ concentration of peppermint EO (5.3%) and 800 μL L^−1^ concentration of thyme (15.8%) to decrease the infection on detached strawberry leaves compared to inoculated control 4 DAI (Table 2). Sage EO had no positive influence on infection spread.

## 3. Discussion

There is a growing interest in EO, and their components due to their volatility, relative safety, and wide acceptance by consumers, as well as their ecological and biodegradable properties. For our study, we selected *T. vulgaris*, *S. officinalis,* and *M. piperita* EO, and analyzed the chemical composition, antifungal activities and EO effect on detached strawberry leaves, to assess the feasibility of using EO as biocontrol agents in disease control. EO from thyme, sage, peppermint presented noticeable antifungal activity against *C. acutatum* in vitro. 

Our research data confirmed that the chemical composition of tested EO is in line with what is already have been described in the literature [16,17,19,20,26,29,30,31,32,33,34]. Oliveira et al. [16] reported that thymol and *p*-cymene were abundant components in identified 28 compounds of thyme EO. Kim et al. [19] stated that the most common compound differed according to the area. These results were equivalent to our study. In an investigation conducted by Duduk et al. [31], thyme EO showed good antifungal efficacy against *C. acutatum* on strawberry fruit. In our study, thyme EO inhibited *C. acutatum* mycelial growth in vitro above 200 μL L^−1^. This suggests that the antifungal effect presence of the dominant components of EO, as main activity carriers. Palfi et al. [17] reported that thyme, sage, and peppermint EO totally inhibited the mycelial growth of *F. oxysporum* in vitro; however, sage EO had a low inhibitory effect against *B. cinerea*. Oliveira et al. [32] observed that 5 μL/mL peppermint EO showed 100% MGI on all tested *Colletotrichum* stains. In our research peppermint, EO highest antifungal activity reached 1600 μL L^−1^ (88%). In comparison, Oliveira et al. [32] identified 26 different constituents of peppermint EO, and the dominant compounds were: menthol (41.34%), isomenthone (23.47%), cis-menthone (23.47%), while in our studies 7.95, 12.81, 44.56%, respectively. Hong et al. [6] evaluated plant EO component menthone antimicrobial activity during conidial germination and mycelial growth of *C. gloeosporioides*. Menthone demonstrated relatively low antifungal activity on conidia germination and pepper fruits. After reviewing the previous literature [6,24,32], the assumption could be that the higher content of menthol in the essential oil has a higher inhibitory effect against *Colletotrichum* spp. The antifungal properties of sage EO primarily affect the main abundant constituents α,β-thujone, camphor, and eucalyptol [20,29,33,34]. In our research, the antifungal activity of sage EO against *C. acutatum* achieved the highest effect of 88.14% at 1800 μL L^−1^. In comparison, Yilmaz et al. [34] studied, that the application of sage EO resulted in slight inhibition on mycelial growth of *C. gloeosporioides* in fumigation bioassay and contact bioassay in vitro (solid media) and in vivo (apple) conditions. These results supported our findings, where the predominant compounds of sage EO, showed antifungal effect against *C. acutatum* but did not suppress it. Chemical compounds found in lower amounts in EO may also influence its antifungal properties.

However, to the best of our knowledge, no investigations have been previously performed on the antifungal effect of thyme, sage, and peppermint EO on detached strawberry leaves against *C. acutatum.* The present research, investigated EO result on the detached strawberry leaves assay indicated a less positive effect of reducing the spread of anthracnose infection. 1000 μL L^−1^ concentration of peppermint EO (15.8%) and 800 μL L^−1^ concentration of thyme decreased (5.3%) the infection on strawberry leaves. A higher concentration of this EO’s should be investigated, to obtain greater efficiency. Plants effect as volatile compounds on EO may induce a stressful environment on the surface of strawberry leaves [11,35].

In summary, examining various EO and their concentrations in vitro exhibited promising prospects against strawberry anthracnose; however, the antifungal effect on detached strawberry leaves was low.

## 4. Materials and Methods

### 4.1. Essential Oil Extraction

The essential oils (EO) were extracted by Clevenger-type hydro-distillation. EO obtained from common thyme (*Thymus vulgaris* L.), common sage (*Salvia officinalis* L.), peppermint (*Mentha piperita* L.) was chosen for the determination of antifungal activity against strawberry pathogen *C. acutatum* at different concentrations. Plants for essential oils extraction were obtained from Lithuanian Research Centre for Agriculture and Forestry (LAMMC) Institute of Horticulture (IH) experimental fields (55.081052, 23.806630).

### 4.2. Identification of the Essential Oils Chemical Composition

Volatile compounds of essential oils were established by gas chromatography - mass spectrometry (GC-MS). The analysis was executed on GC-2010Plus/GCMS-QP2010 Ultra system (Shimadzu, Kyoto, Japan) equipped with Rxi-5MS capillary column (33 m × 0.25 mm; 0.25 μm) (Restek, Bellefonte, PA, USA), as defined in previous studies [11,12].

### 4.3. C. acutatum Isolates

The research carried out at the Laboratory of Plant Protection, LAMMC IH in 2017–2020. To obtain pure single-spore culture isolation from *C. acutatum* (infected strawberry ‘Deluxe’ fruits) performed. The selected isolates cultured on potato dextrose agar (PDA) at 25 °C for 7 days. The isolates initially identified by morphological traits typical of the colonies [36] and confirmed by PCR as *C. acutatum* by Moreira et al. [37]. 

### 4.4. Essential Oils Antifungal Activity In Vitro 

To evaluate antifungal activity against *C. acutatum* 50–1000 µL L^−1^ concentrations of thyme EO and 200–1800 µL L^−1^ sage and peppermint EO were used. Different concentrations of pure essential oil were added to cool at 45 °C PDA. Four repetitions with four replications were carried out. Petri plates inoculation was with 5 mm *C. acutatum* mycelial plugs of 7-day old fungus. The mycelium was put upside down (mycelia side) in Petri’s center containing PDA with different EO concentrations. The Petri plates were incubated at 25 ± 2 °C in the dark. The control treatments were oil-free. 

The diameter (mm) of *C. acutatum* colony growth measured in two directions after 2, 4, and 7 days after inoculation (DAI). The mean of colony growth diameter used for mycelial growth inhibition calculations. The mycelial growth inhibition (MGI) (%) was determined using the formula:

Mycelial growth inhibition (%) = (*C* − *T*)/*C* × 100,

*C* is the mycelium diameter of the pathogen colony in control Petri dish, mm; *T*—mycelium diameter of the pathogen colony in the essential oil-treated Petri dish, mm [11,38,39]. Minimal inhibitory concentration (MIC) was determined as EO concentration with 100% MGI [11].

### 4.5. Essential Oils Antifungal Activity on Detached Strawberry Leaves

The essential oils inhibitory effect evaluated on detached strawberry cultivar ‘Deluxe’ leaves. Healthy strawberry leaves, consisting of three petiole leaflets, without any visible symptoms of the disease were soaked in 70% ethanol solution for 3 min and rinsed 4–5 times with sterile distilled water (SDW). Each leaf was placed in a Petri dish with 5 mL of SDW. Detached strawberry leaves were sprayed with essential oils (800 and 1000 µL L^−1^), then were wounded with sterile needle and a 9-mm plug of 7-day-old *C. acutatum* was placed on the wound. Incubation was carried out at 25 ± 2 °C in the dark for 7 days. The 16 leaves used in treatment; the experiment repeated three times with four replicates. The control treatments were not sprayed with EO but inoculated with *C. acutatum* (Inoculated control). The treatments antifungal activity is assessed by the disease severity (DS) and disease reduction (DR) in the leaves. 

Disease severity (DS) of each inoculated plant leaf assessed at 4 and 7 DAI by calculating the percentage of leaf area affected: (1) 0%—no visible infection, (2) 5%, (3) 10%, (4) 20% and (5) 50% or more area of leaf infected [40,41,42].

*DS* (%) = (((0 × *P*0) + (1 × *P*1) + (2 × *P*2) + (3 × *P*3) + (4 × *P*4) + (5 × *P*5))/*N* × *G*) × 100

where *P*0 to *P*5 is the total number of the evaluated leaves in each corresponding scale, *N*—total number of leaves, *G*—number of maximum grades observed in scale [42].

*DR* (%) = (*Xc* − *Xt*)/*Xc* × 100

where *Xt* is the mean of *DS* per treatment, and *Xc* is the mean of *DS* in the inoculated control [43].

### 4.6. Statistical Analysis

The SAS Enterprise Guide 7.1 program (SAS Institute Inc., Cary, NC, USA) used for the analysis of experimental data. The analysis of variance (ANOVA) processed, and Duncan’s multiple range test (*p* < 0.05) used for the comparison of obtained means. The standard error (SE) in the figures marked as an error bar estimated for isolates growth rates.

## 5. Conclusions

The essential oil of thyme, containing dominant compound thymol, showed total inhibition against *C. acutatum* in vitro. Peppermint and sage EO containing predominant components menthone, isomenthone, and thujone, camphor, respectively, showed significant antifungal activity at the highest concentrations. *C. acutatum* mycelial growth on detached strawberry leaves was slightly reduced by applying thyme EO and more suppressed by peppermint EO at tested concentration. Sage EO did not influence the spread of *C. acutatum* on detached strawberry leaves. The detached strawberry leaves assay revealed that the investigated essential oils were not equally effective and needed further investigations with higher concentrations.

## Figures and Tables

**Table 1:** Composition of the essential oil compounds of thyme (*T. vulgaris*), sage (*S. officinalis*), and peppermint (*M. piperita*).

Essential Oils	*Thymus vulgaris*		*Salvia officinalis*		*Mentha piperita*	
Component	PA ^1^ (%)	RT ^2^	PA (%)	RT	PA (%)	RT
Tricyclene			0.16	6.388		
α-thujene	1.06	6.488	0.17	6.503		
α-pinene	1.09	6.667	3.23	6.689	0.73	6.682
Camphene	0.38	7.079	5.5	7.079		
Sabinene			0.12	7.702	0.55	7.701
β-pinene	0.43	7.779	2.65	7.798	0.87	7.795
1-octen-3-ol	0.94	7.888				
Myrcene	2.42	8.139	1.18	8.147	0.48	8.147
3-octanol					0.21	8.344
α-phellandrene	0.32	8.517				
δ-3-carene	0.15	8.673				
α-terpinene	2.52	8.863	0.15	8.871	0.47	8.869
p-cymene	16.95	9.157	0.18	9.107	0.47	9.1
Limonene	0.81	9.228	1.21	9.237	0.92	9.219
Eucalyptol	1.88	9.285	10.33	9.3	3.35	9.283
cis-β-ocimene	0.11	9.432	0.23	9.444	0.16	9.442
γ-terpinene	10.81	10.092	0.32	10.053	0.73	10.053
4-pentenyl butyrate	0.15	10.214				
cis-sabinene hydrate	1.04	10.328	0.16	10.351	2.51	10.352
Terpinolene	0.17	10.876	0.32	10.894	0.2	10.895
trans-sabinene hydrate					0.21	11.257
Linalool	3.47	11.255	0.47	11.339	0.33	11.291
α-thujone	0.6	11.39	25.8	11.507		
β-thujone	0.21	11.708	8.65	11.775		
Isothujol			0.17	12.371		
cis-p-menth-2-en-1-ol					0.19	11.939
trans-p-menth-2-en--1ol + trans-Sabinol					0.22	12.548
Menthone					44.56	12.964
Isomenthone					12.81	13.2
Camphor	0.74	12.619	20.46	12.616		
trans-pinocamphone			0.14	12.98		
Borneol	0.75	13.208	4.37	13.223		
δ-terpineol + borneol					0.24	13.24
cis-pinocamphone			0.2	13.385		
Menthol					7.95	13.473
Terpinen-4-ol	1.05	13.483	0.28	13.494	2.58	13.549
α-terpineol	0.25	13.999	0.22	13.879	0.32	13.916
Thymol methyl ether	0.61	15.01				
Carvacrol methyl ether	0.63	15.272				
Carvone Z, dihydro					0.31	14.039
Myrtenol			0.28	14.056		
cis-3-hexenyl-isovalerate					0.11	15.072
Pulegone					10.74	15.282
Bornyl acetate			1.39	16.431		
Thymol	41.35	16.984				
Carvone					0.45	15.346
Carvacrol	2.57	17.123				
trans-sabinyl acetate + thujyl acetate			0.1	16.597		
Piperitone					1.65	15.636
Caryophyllene E	1.86	20.018	2.84	20.013	0.75	20.004
Menthyl acetate					1.41	16.639
α-humulene	0.17	20.859	3.25	20.872		
Geranyl propanoate	0.12	21.212				
γ-cadinene	0.14	22.324				
δ-cadinene	0.22	22.517				
Piperitenone					0.36	17.97
β-elemene					0.34	19.258
Germacrene D					0.79	21.536
Bicyclogermacren					0.2	21.908
Caryophyllene oxide	0.31	24.066	0.19	24.059		
Viridiflorol	0.11	24.337	3.03	24.363		
Humulene epoxide II			0.26	24.796		
Manool			0.74	32.227		
α-muurolol						
Unknown	0.49					
Squalene	1.19	36.912				
Di-n-octyl phthalate	0.13	37.496			0.2	37.486
Other ^3^	1.64		1.41		1.68	
Total Identified	99.33		99.94		99.94	

^1^ PA—peak area. ^2^ RT—retention time. ^3^ The compounds that were less than 0.1% of the quantity of the essential oil.

**Table 2:** The disease severity and reduction in anthracnose regarding strawberry cultivar ‘Deluxe’ by different essential oils concentrations at 4 days after inoculation. Means *n* = 4 ± SE.

Treatments	Disease Severity (%)	Disease Reduction (%)
Inoculated control	79.2 ± 0.2	n.a.*
*Thymus vulgaris* 800 μL/L	75 ± 0.3	5.3
*Salvia officinalis* 1000 μL/L	80.6 ± 0.2	0
*Mentha piperita* 1000 μL/L	66.7 ± 0.2	15.8

* n.a.—not applicable.

## Supplementary Materials

The following are available online at https://www.mdpi.com/2223-7747/10/1/114/s1.
