# Supplementary material for: The Use of Essential Oils from Thyme, Sage and Peppermint against Colletotrichum acutatum
Source: Plants (Basel). 2021 Jan 8;10(1):114. doi: 10.3390/plants10010114 (PMC7827828; doi:10.3390/plants10010114)
Supplement: Supplementary file 1 [file plants-10-00114-s001.zip › Table S1.docx]

**Table S1.** The sage and peppermint essential oils on mycelial growth (mm) of *C. acutatum.* Results are presented as mean ± SE (*n* = 4)

| Sage EO | | | | | Peppermint EO | | | |
| --- | --- | --- | --- | --- | --- | --- | --- | --- |
| **Days** | **4 DAI** |  | **7 DAI** |  | **4 DAI** |  | **7 DAI** |  |
| **Control** | 31.9 | ±0.1 | 49.1 | ± 0.2 | 36.4 | ± 0.1 | 49.7 | ± 0.1 |
| **200 µl L^-1^** | 32.2 | ± 0.2 | 45.6 | ± 0.3 | 36.5 | ± 0.0 | 49.4 | ± 0.1 |
| **400 µl L^-1^** | 38.9 | ± 0.0 | 55.8 | ± 0.0 | 34.9 | ± 0.1 | 48.5 | ± 0.1 |
| **600 µl L^-1^** | 32.3 | ± 0.2 | 47.0 | ± 0.2 | 32.7 | ± 0.1 | 45.3 | ± 0.1 |
| **800 µl L^-1^** | 31.5 | ± 0.2 | 45.1 | ± 0.3 | 29.1 | ± 0.1 | 41.0 | ± 0.1 |
| **1000 µl L^-1^** | 23.4 | ± 0.2 | 38.8 | ± 0.2 | 22.3 | ± 0.1 | 34.9 | ± 0.1 |
| **1200 µl L^-1^** | 13.0 | ± 0.1 | 25.1 | ± 0.1 | 9.9 | ± 0.0 | 20.1 | ± 0.0 |
| **1400 µl L^-1^** | 11.8 | ± 0.1 | 24.8 | ± 0.1 | 7.3 | ± 0.0 | 15.9 | ± 0.1 |
| **1600 µl L^-1^** | 9.9 | ± 0.1 | 23.0 | ± 0.1 | 3.9 | ± 0.0 | 15.1 | ± 0.0 |
| **1800 µl L^-1^** | 3.8 | ± 0.2 | 18.6 | ± 0.1 | 7.1 | ± 0.0 | 19.0 | ± 0.1 |
| **LSD** | 2.73 |  | 3.58 |  | 1.50 |  | 2.44 |  |

LSD= least significant difference (p < 0.05)
