# Supplementary material for: The Use of Essential Oils from Thyme, Sage and Peppermint against Colletotrichum acutatum
Source: Plants (Basel). 2021 Jan 8;10(1):114. doi: 10.3390/plants10010114 (PMC7827828; doi:10.3390/plants10010114)
Supplement: Supplementary file 1 [file plants-10-00114-s001.zip › Table S2.docx]

**Table S2.** The thyme essential oil on mycelial growth (mm) of *C. acutatum*. Results are presented as mean ± SE (n = 4)

| Thyme EO | | | | |
| --- | --- | --- | --- | --- |
| **Days** | **4 DAI** |  | **7 DAI** |  |
| **Control** | 28.1 | ± 0.1 | 47.2 | ± 0.2 |
| **50 µl L^-1^** | 13.3 | ± 0.0 | 23.1 | ± 0.0 |
| **100 µl L^-1^** | 0.0 | ± 0.0 | 0.0 | ± 0.0 |
| **150 µl L^-1^** | 3.8 | ± 0.1 | 4.8 | ± 0.1 |
| **200 µl L^-1^** | 0.0 | ± 0.0 | 0.0 | ± 0.0 |
| **250 µl L^-1^** | 0.0 | ± 0.0 | 0.0 | ± 0.0 |
| **600 µl L^-1^** | 0.0 | ± 0.0 | 0.0 | ± 0.0 |
| **800 µl L^-1^** | 0.0 | ± 0.0 | 0.0 | ± 0.0 |
| **1000 µl L^-1^** | 0.0 | ± 0.0 | 0.0 | ± 0.0 |
| **LSD** | 1.01 |  | 2.29 |  |

LSD= least significant difference (p < 0.05)
