# Supplementary figures and images for: The Use of Essential Oils from Thyme, Sage and Peppermint against Colletotrichum acutatum
Source: Plants (Basel). 2021 Jan 8;10(1):114. doi: 10.3390/plants10010114 (PMC7827828; doi:10.3390/plants10010114)

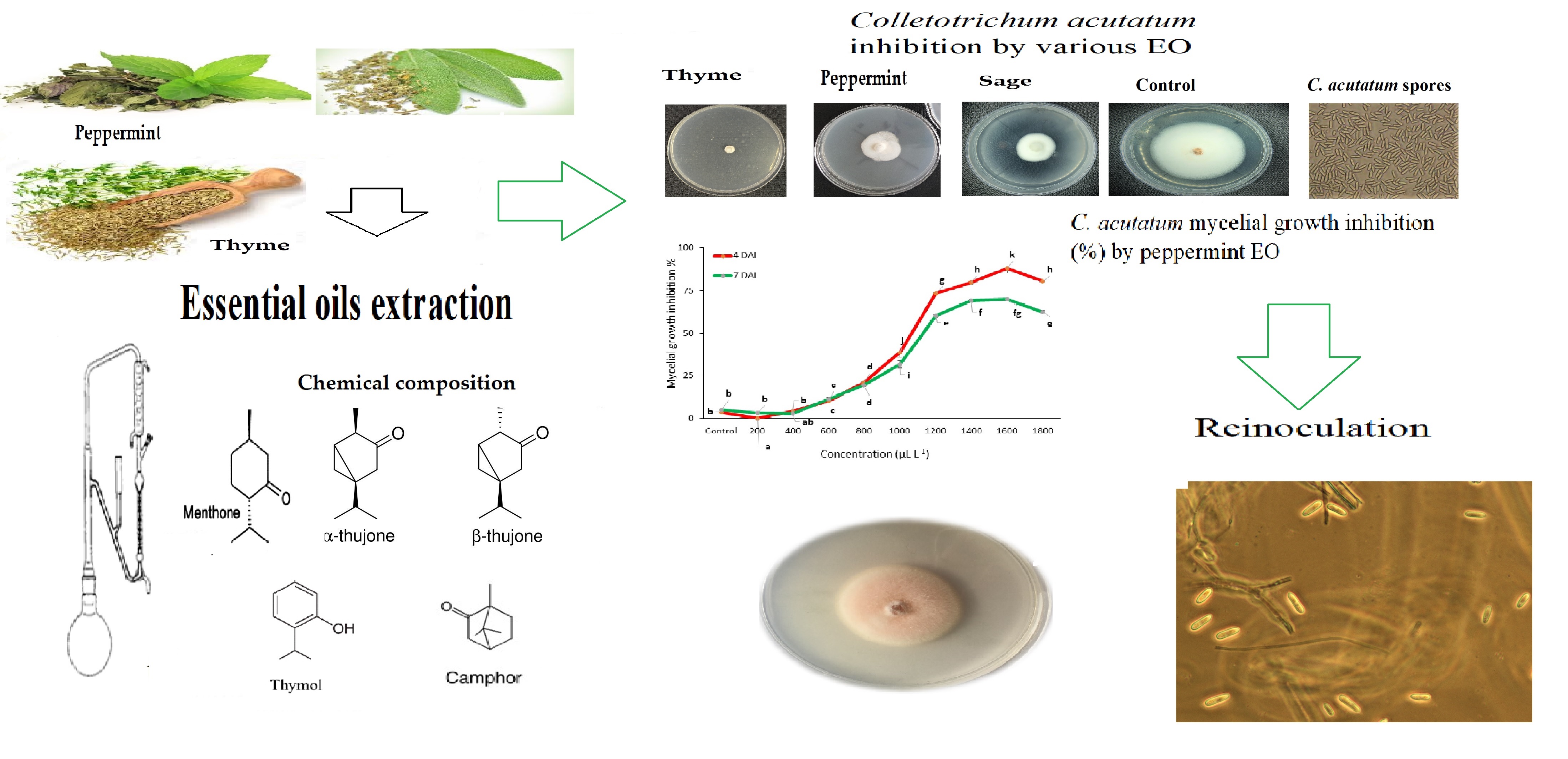

Supplement: Supplementary file 1 [file plants-10-00114-s001.zip › Figure S1.jpg]

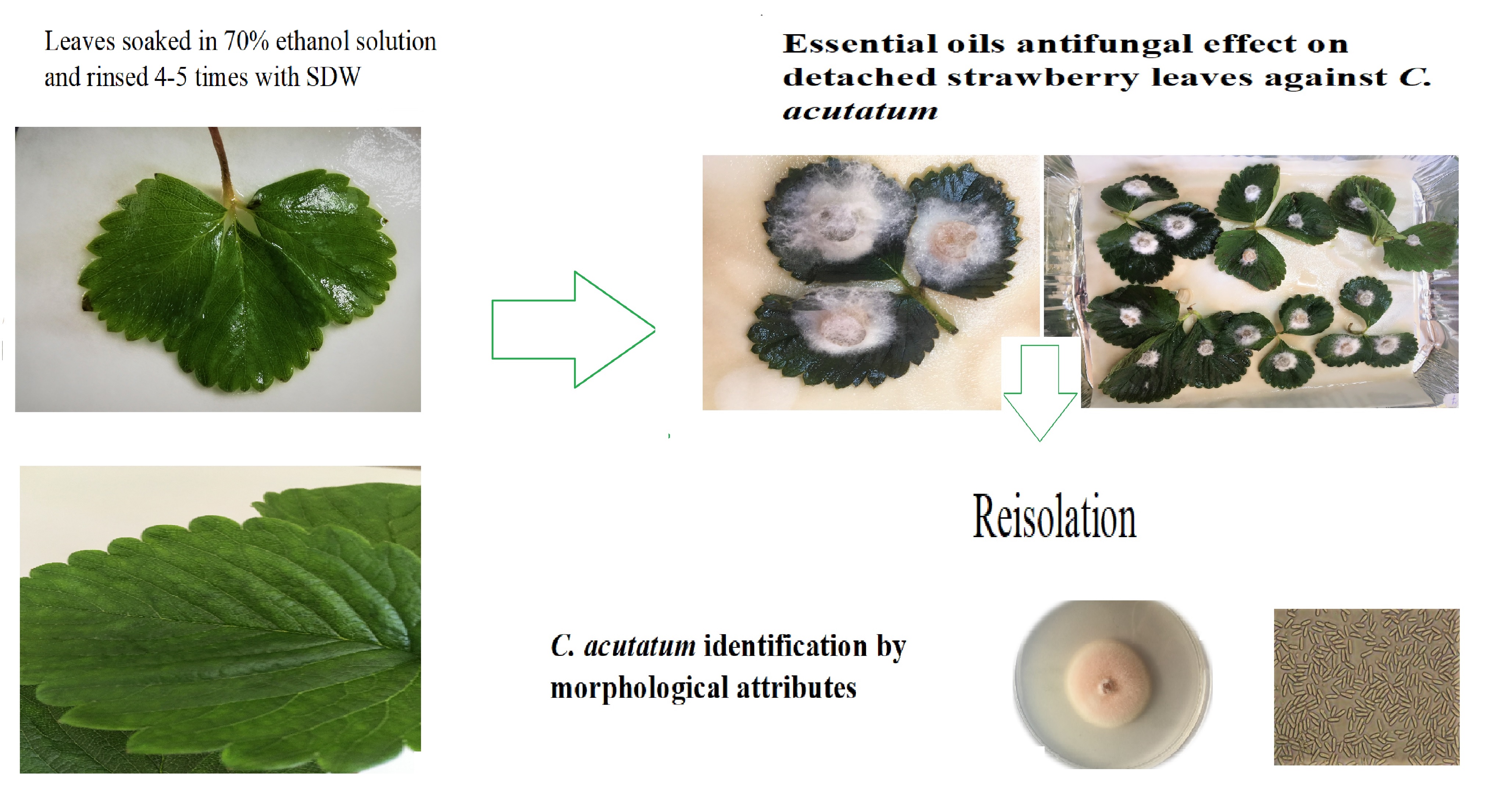

Supplement: Supplementary file 1 [file plants-10-00114-s001.zip › Figure S2.jpg]
